# Modelling of retinal vasculature based on genetically tuned parametric L-system

**DOI:** 10.1098/rsos.171639

**Published:** 2018-05-09

**Authors:** Seyed Mohammad Ali Aghamirmohammadali, Ramin Bozorgmehry Boozarjomehry, Mohammad Abdekhodaie

**Affiliations:** Chemical and Petroleum Engineering Department, Sharif University of Technology, Azadi Av., Tehran, Iran

**Keywords:** retinal vasculature, parametric L-system, L-system inverse problem, mathematical morphology

## Abstract

Structures of retinal blood vessels are of great importance in diagnosis and treatment of diseases that affect the eyes. Parametric Lindenmayer system (L-system) is one of the powerful rule-based methods that has a great capability for generating tree-like structures using simple rewriting rules. In this study, a novel framework, which can be used to model the retinal vasculature based on available images, has been proposed. This framework presents a solution to special instance of a general open problem, the L-system inverse problem, in which L-system rules should be obtained based on images representing a particular tree-like structure. In this study, genetic algorithm with a novel objective function based on feature matching and an L-system grammar comparison has been used along with nonlinear regression to solve the parametric L-system inverse problem. The resulting L-system growth rules have been employed to predict inaccessible vascular branches. Graphical and quantitative comparison between model results and target structures of different case studies reveals that the proposed framework can be used to generate the structure of retinal blood vessels accurately. Even in the cases lacking sufficient image data, it can provide acceptable predictions.

## 1. Introduction

Blood vessels play a critical role in diagnosis and treatment of many diseases such as cancer, hypertension, atherosclerosis and diabetes [1]. Over the past two decades, many works were devoted to study vasculogenesis [2], angiogenesis [3] and blood vessel morphology [4]. These studies have often focused on physiology [5], modelling [6], and simulation of the morphology and behaviour of blood vessels [7].

Morphological analysis of blood vessels can be used to identify conditions and diseases [8]. Also, a reliable vascular structure model is necessary to investigate haemodynamic parameters and drug delivery conditions in the circulatory system [9]. Image data is a useful tool to extract blood vessel morphological properties such as length, width, tortuosity and branching pattern. Retinal fundus image is one of the most important data for diagnosis and treatment of diseases that change the morphological and functional characteristics of blood vessels [8]. This is due to the fact that retina is the only organ whose blood vessels can be visualized non-invasively and directly *in vivo*. Retinal blood vessel extraction and modelling has found applications in automatic early disease diagnosis and monitoring such as diabetic retinopathy, hypertensive retinopathy, and branch retinal vein occlusion [10], evaluation and optimization of eye nutrient, oxygen, and drug delivery [11,12], as well as examination of the factors affecting retinal vascularization and angiogenesis [13].

Previous works on retinal blood vessel segmentation and modelling can be mainly divided into three categories: image processing methods, mathematical modelling methods and rule-based methods.

Image processing is the most common tool for vessel extraction. Various techniques were developed for retinal fundus image analysis and segmentation [14]. Kirbas & Quek [15] have divided image processing methods for retinal blood vessel segmentation into five main categories: (i) pattern recognition techniques, (ii) model-based approaches, (iii) tracking-based approaches, (iv) artificial intelligence-based approaches, and (v) tube-like object detection approaches. Performance of each method can be evaluated and compared with indexes such as sensitivity, specificity and accuracy. The results of image processing methods are generally in good agreement with source images which typically have an accuracy of more than 0.8, a sensitivity of more than 0.6 and a selectivity of more than 0.9 [14]. However, image processing techniques are quite sensitive to image quality and resolution, and they are not able to estimate or reproduce low resolution branches. Furthermore, these techniques can only be used for identification of the vasculature. However, they are not capable of predicting the changes in the structure of vasculatures resulting from various diseases. In addition, time-variant modelling of angiogenesis or vasculogenesis needs other techniques such as mathematical modelling or rule-based modelling methods.

Extensive experimental works and mathematical modelling were conducted to investigate blood vessel formation, sprouting, and growing processes [16,17]. Differences between the models can be categorized in terms of problem scale, affecting factors, assumptions and model complexity. Various factors that include vascular endothelial growth factor, platelet-derived growth factor, fibronectin, tumour angiogenesis factor, matrix metalloproteinases and angiopoietin-1 are taken into the consideration to model retinal vasculature [18]. On the other hand, different modelling approaches such as continuum-based modelling, discrete modelling, stochastic modelling and deterministic modelling were also applied in this regard [19–21]. The mathematical modelling is based on the basic knowledge of vasculogenesis and angiogenesis physiology. The complex nature of blood vessel formation and growth, as well as limited knowledge of the interaction between effective factors, are the main barriers for mathematical modelling. It should also be mentioned that assumptions used in the various models along with unknown parameters result in an inconsistency between morphological properties of simulated vascular networks and image data.

The common rule-based modelling approaches are diffusion-limited aggregation (DLA) and Lindenmayer system (L-system). Since Witten & Sander [22] proposed DLA, some efforts were conducted to model biological structure, such as vasculature network [23,24]. DLA is based on Brownian motion and aggregation of particles to form tree-like fractal structure. Family *et al*. [25] showed that fractal dimension in the normal retina is approximately equal to that in the results from DLA modelling. However, structures derived from DLA modelling do not match the morphological information obtained based on the image data due to the stochastic nature of the DLA approach.

Originally, Lindenmayer [26] developed L-system to model cell division in multicellular organisms. Prusinkiewicz & Lindenmayer [27] discussed the evolution of simple L-system to parametric, stochastic, context-sensitive, and bracketed L-system, which can be used to simulate complex structures. Zamir [28] used parametric L-system to model arterial branching by applying a pre-defined rule to create tree-like fractal structures. He also introduced an algorithm based on bifurcation index [28] and Murray's law [29,30] to calculate morphological properties such as length, diameter and the angles in each branch. He showed that a constant bifurcation ratio results in a uniform fractal structure that is rarely observed in the physiological system. To alleviate this, he used random bifurcation ratio for each parameter calculation to produce more realistic structures. Liu *et al*. [31] employed the constant bifurcation index and a random multiplier in calculations to have a more accurate pattern. Hichem & Malek [32] introduced an automatic computer-aided algorithm to determine branch crossovers and endpoints for diameter calculations. They also tried to test the prediction capability of parametric L-system with modelling of blood vessels that were smaller than image resolution. Owing to the complexity of unknown rules and parameters, none of those mentioned simulated structures was matched perfectly with image data.

Kókai *et al*. [33] presented grammatical retina description with evolutionary algorithms to model retinal vasculature structure automatically. They used genetic algorithm (GA) as an optimization tool to achieve grammatical representation of image data. However, the output of their algorithm is merely a final alphabetic string without any rewriting rule which is based on image resolution and cannot be used to predict inaccessible branches in the obtained structure.

In the present work, a novel method was proposed to model, simulate and analyse the retinal blood vessels. The proposed method is based on parametric L-system and GA for rule extraction and parameter estimation of the model which regenerates retinal blood vessels. In fact, such a problem is formulated as a special case of an open problem called parametric L-system inverse problem [34]. In the current work, an algorithm is proposed for the first time to solve this problem and find the parametric growth rules of a target structure. For this purpose, a novel hybrid fitness function was introduced based on which the modelling of retinal vasculature is formulated as a mixed integer nonlinear programming (MINLP) problem which was solved by GA to that purpose. Also using obtained L-system model to reconstruct inaccessible branches as prediction capability has been investigated.

The rest of the paper is organized as follows. Lindenmayer system is briefly described in §2, after which the application of L-system in the retinal vasculature modelling is explained in §3. Since there are lots of parameters involved in the growth rule of the parametric L-system, and they should all be estimated efficiently, the formulation of such a problem as an MINLP is elaborated in §4. The optimization technique which is a newly developed variant of GA is introduced in §5, which is then followed by §6 in which the proposed method is described. Section 7 contains the results obtained by the proposed framework for retinal vasculatures of two selected benchmarks.

## 2. L-system

The main concept of L-system is to use simple objects to generate complex objects by applying a set of rewriting rules. Each L-system contains an initiator string called axiom, and a set of rewriting rules.

Context-free and deterministic L-system, called DOL-system, can be shown as an ordered triplet G=⟨V,ω,P⟩. If *V* is the alphabet of the L-system, $V^{*}$ and $V^{+}$ are the set of all words and non-empty words over *V*, respectively. $\omega \in V^{+}$ is a non-empty word called axiom and $P\subset V\times V^{*}$ is a finite set of rewriting or growth rules. A growth rule is written as ‘predecessor→successor’. The predecessor is iteratively replaced by the successor in the resulting string. In deterministic L-system, there is exactly one successor for each predecessor [27].

In order to take into account the impact of the external factors on the growth and evolution of the object structure, Lindenmayer [35] proposed parametric L-system. Parametric L-system is considered as an ordered quadruplet, G=⟨V,Σ,ω,P⟩, where Σ is the set of formal parameters [27]. Usually, parameters represent the length and angle dimensions which can be varied in each rewriting step through nonlinear or linear functions. Also, bracketed L-system was presented to model tree-like structures [26].

Turtle interpretation of string has been proposed to convert L-system strings to graphical structures [36]. A state of the turtle was considered as (x,y,α), where (*x*,*y*) demonstrate the Cartesian coordinates of turtle position and the angle *α* represent turtle orientation. The step length and the step angle were considered to be *d* and *δ*, respectively [27]. L-system symbols which were used in this work for retinal vasculature modelling are shown in table 1.

**Table 1.** Turtle interpretation of symbols.

symbols	interpretation
*F*(*d*), *A*(*d*) and *B*(*d*)	move forward and change the state to (x+d cosα,y+d sinα,α)
+(δ)	turn left and change the state to (x,y,α+δ)
−(δ)	turn left and change the state to (x,y,α−δ)
[	push the state on the stack
]	pop the state from the stack, move to (x,y,α)
∅	null operator

According to the L-system model, the axiom is going through the changes described by the growth rules. At the end of each growth cycle, the obtained string acts similar to the axiom. This procedure continues until the stage at which none of the growth rules can induce any change at the end of cycle string, or it gets to the maximum desired growth cycle. The following example is provided to clarify the L-system rewriting and interpretation procedure.

**Figure 1.**
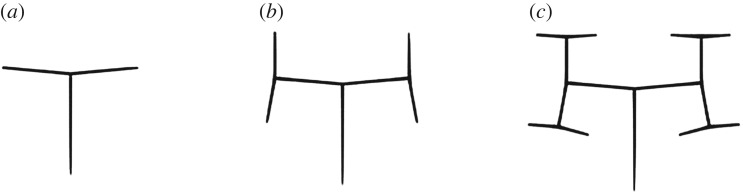
Interpretation of L-system defined in example 2.1: (*a*) growth cycle = 1, (*b*) growth cycle = 2, and (*c*) growth cycle = 3 [27].

Example 2.1. The following parametric L-system [27] results in the structures shown in figure 1:

2.1 $$\begin{array}{l} \omega \;:\;A(100,90^{\circ })\\ p_{1}:A(Z)\to F(Z)\left[+A\left(\frac{Z}{R}\right)\right]\left[-A\left(\frac{Z}{R}\right)\right]\\ R=1.5,\;\delta =85^{\circ } \end{array}\}$$

2.2 $$\begin{array}{l} \text{growth }\;\text{cycle }0\;A(100)\\ \text{growth}\;\text{ cycle }1\;F(100)[+A(66.6)][-A(66.6)]\\ \text{growth }\;\text{cycle }2\;F(100)[+F(66.6)[+A(44.4)][-A(44.4)]][-F(66.6)[+A(44.4)][-A(44.4)]]\\ \text{growth }\;\text{cycle }3\;F(100)\\ \;\;\;\;\;\;\;[+F(66.6)[+F(44.4)[+A(29.6)][-A(29.6)]][-F(44.4)[+A(29.6)][-A(29.6)]]]\\ \;\;\;\;\;\;\;[-F(66.6)[+F(44.4)[+A(29.6)][-A(29.6)]][-F(44.4)[+A(29.6)][-A(29.6)]]]. \end{array}\}$$

## 3. Application of L-system in retinal vasculature modelling

Parametric L-system can be used to model non-uniform tree-like structures such as retinal blood vessels. It could be shown that every tree-like structure can be represented as an L-system string. However, obtaining axiom and rewriting rules from a complex string is a complicated problem which is known as L-system inverse problem. The variant nature of string and parameters in L-system which can be changed during each rewriting growth cycle and also tracking these changes make this problem difficult to tackle.

**Figure 2.**
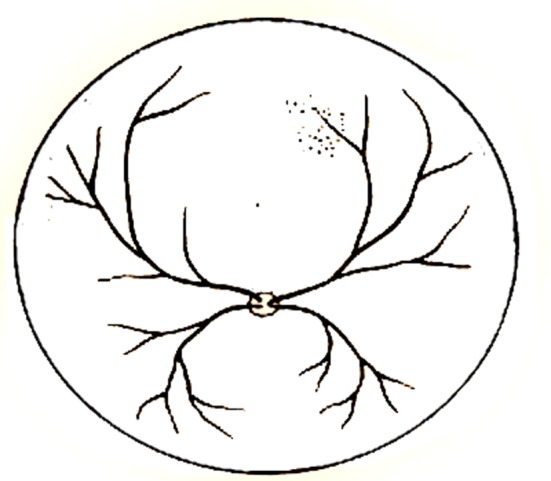
Preprocessed retina image presented by Kókai *et al*. [33].

Kókai *et al*. [33] converted a segment of preprocessed retina image shown in figure 2 to an L-system string.

The following string was given by Kókai *et al*. [33] to describe the structure of the top-right quarter of retina image shown in figure 2:

3.1 $$\begin{array}{rl} & F(37)[+(38)F(16)[+(27)F(11)+(14)F(12)+\\ & \;(6)F(19)[+(47)F(19)][-(6)F(17)]][-(14)F(22)+(37)F(19)[-(6)F(12)+\\ & \;(34)F(18)+(32)F(14)][-(43)F(24)]]][F(37)[-(7)F(8)+(43)F(24)][-(14)F(26)]], \end{array}$$

in which *F*(*d*) represents a line of length *d* and ±(*δ*) denotes rotation by angle *δ*.

However, their work was only limited to finding a final L-system string while the growth rules required for producing this string were not provided. Growth rules can be applied to predict zones with low resolution, high noise or out of image scope. Also, segmentation of overlaid branches with different origins will be possibly more reliable.

Some efforts have been conducted to find rewriting rules from a non-biological structure. Koza [37] used genetic programming to achieve rewriting rules for non-parametric L-system using a pattern matching approach as the fitness function. Santos & Coelho [38] developed a method based on string growth analysis that can be used to convert a non-parametric string to its original L-system. Št'ava *et al*. [34] introduced a new method based on recognizing similar elements in images and assigning L-system alphabets to them. However, similar elements and uniform structures are rarely seen in biological structures. All of the mentioned methods are rooted in the concept of solving a non-parametric L-system inverse problem. Here, a new method is proposed to obtain parametric L-system from image data to simulate non-uniform structures such as retinal vasculature. This method was implemented in a C++ object-oriented platform [39] and used to solve two benchmarks to show its performance along with its flexibility.

## 4. Problem statement

The modelling of retinal vascularization can be stated in a more general and abstract manner as follows.

For a given tree-like target structure image (such as retinal blood vessel), the growth rules of an L-system with a set of general parameters that can provide a good approximate of the target structure are desired.

This problem can be formulated as the following optimization problem:

4.1 $$\begin{array}{c} \min f(\text{St}{\text{r}}_{t},\text{St}{\text{r}}_{s})\\ \text{subject to}:\\ \text{St}{\text{r}}_{s}=G\langle \omega ,p_{1\ldots n},M_{m,n}^{b}\rangle , \end{array}\}$$

where *f* calculates morphological differences between simulated structure ($\text{St}{\text{r}}_{s}$), resulting from L-system, and target structure ($\text{St}{\text{r}}_{t}$) obtained from fundus images. In fact, $\text{St}{\text{r}}_{s}$ is obtained from the L-system supposed to model the tree-like structure of the retinal vasculature. Considering *M_m_* as a sequence of L-system parametric symbols defined as below, equation (4.2) represents such a model ($G\langle \omega ,p_{1\ldots n},M_{m,n}^{b}\rangle$) in its general form:

4.2 $$\begin{array}{l} \omega :T_{0}^{\prime }(z_{0},\theta_{0})\\ p_{1\ldots n}:T_{1,1\ldots n}^{\prime }(Z,\Theta )\to M_{m,n}^{0}[M_{m,n}^{1}][M_{m,n}^{2}]\\ M_{m,n}^{b}={\left(D^{b}(g^{b}(Z,\Theta ))T^{b}(f^{b}(Z,\Theta ))\right)}_{1\ldots m,1\ldots n} \end{array}\}$$

where

$$\begin{array}{ll} & T\in \{F,A,B,\emptyset \},\\ & T^{\prime }\in \{F,A,B\},\\ & D\in \{+,-,\emptyset \},\\ & g(Z,\Theta ),f(Z,\Theta ):\text{functions}\;\text{as}\;\text{the}\;\text{rules}\;\text{parameters}\\ \text{and} & z_{0},\theta_{0}\in R, \end{array}$$

in which ω is axiom, $p_{1\ldots n}$ is rule set, and *b*, *n*, and *m* denote the number of blocks, rules, and alphabets, respectively. g(Z,Θ) and f(Z,Θ) are two general functions that specify the relationship between predecessor and successor parameters. To have a meaningful L-system, axiom and predecessor alphabet (*T*′) could not be null (∅). So, this is why *T*′ was considered as the superset of *T* which shows successor alphabet.

The fitness function and decision variables are both further explained in §§4.1 and 4.2, respectively.

### 4.1. Fitness function

Due to the complexity of parametric L-system inverse problem, definition of an appropriate objective function representing the difference between the modelled structure and the reference retinal vascular image is very important. Here, challenges and weaknesses of various objective functions used in L-system inverse problem modelling are discussed and then a new hybrid objective function is proposed.

#### 4.1.1. Pixel-by-pixel comparison

**Figure 3.**
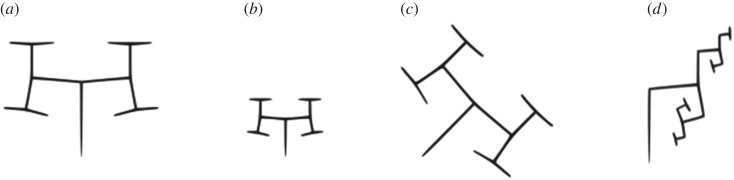
Failure of pixel-by-pixel comparison of (*a*) *A*(100,90°) with (*b*) *A*(50,90°) and (*c*) *A*(100,45°) having the same rules defined in example 2.1 and with (*d*) *A*(100,90°) which has a different rule.

Kókai *et al*. [33] employed pixel-by-pixel comparison as the fitness function to convert image data to an L-system string. Through an illustrative example, it is shown that this fitness function is not useful to drive parametric L-system rewriting rules. Consider the L-system introduced in example 2.1 (figure 3*a*). As is shown in figure 3, changing axiom parameters while keeping the same rules, which leads to the same structure with different scale (figure 3*b*) and orientation (figure 3*c*), pixel-by-pixel comparison gives inappropriate fitness scores to another structure with a different set of rules provided in figure 3*d*.

Figure 3 shows that the main problem with the pixel-by-pixel comparison is that calculated scores do not provide an insight into morphological similarity between two structures.

#### 4.1.2. Image feature matching

Feature matching is one of the most important methodologies to find an object in image data. It is based on detecting important points, called keypoints, in the object template and discovering information about surrounding area of those keypoints, called keypoint description. Descriptors, which contain keypoints description, are compared and matched with target images using some notation of similarity such as Euclidean or Hamming distance of descriptors. So, keypoint detection, description and matching are three main steps to detect object or structure in the image data [40].

Several keypoint detection and description techniques were developed during the past decade [41]. These methods include binary or real-valued descriptors. Many comparative studies were conducted on these methods [42]. Techniques involving binary descriptors require shorter computation times but provide less accurate results. In contrast, methods based on real-value descriptors like scale-invariant feature transform provide very accurate results but with much higher computation times for keypoint detection and description. In this work, the former techniques were selected owing to the faster speed of calculations. Accelerated Segment Test (FAST) [43] and Binary Robust Independent Elementary Features (BRIEF) [44] are well-known keypoint detection and description algorithms respectively. Oriented FAST and rotated BRIEF (ORB) is a keypoint detection and description approach that is a combination of modified FAST and BRIEF algorithms. ORB is rotation and scale invariant. Also, it is robust to data noise and affine transformations [45]. In this study, ORB was employed in both detection and description steps because of its appropriate computational speed and accuracy. Also, owing to its good speed and efficiency for small databases, brute force matcher algorithm was used to match keypoints in this work using Hamming distance calculation in OpenCV library in C++ [46].

Hamming distance is based on executing XOR operations on binary codes. Brute force matcher can compare keypoint descriptors of two images with Hamming distance to correlate the closest ones. Good matches should have a lower distance than a ‘threshold’ which depends on algorithm and problem. Although feature matching can be useful to compare two images, it is not adequate by itself to solve L-system inverse problem.

**Figure 4.**
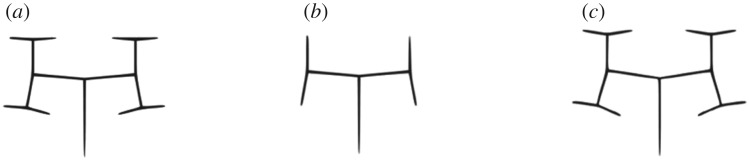
Failure of feature matching in L-systems with the same rules defined in example 2.1 and different parameters: (*a*) 3 growth cycles and *δ* = 85°, (*b*) 2 growth cycles and *δ* = 85° and (*c*) 3 growth cycles and *δ* = 82°.

Example 4.1. To illustrate one of the situations where feature matching fails, consider example 2.1 with different parameters shown in figure 4.

Owing to self-similarity of the structure defined in example 2.1, structure shown in figure 4*b* can be found three times in the one represented in figure 4*a* with different scales. Also, the only difference between structures shown in figure 4*a*,*c* is bifurcation angle that is negligible. Considering figure 4*a* as the target structure, figure 4*c* can be an acceptable solution, but figure 4*b* has a better similarity rank that is not good for optimization purposes.

#### 4.1.3. L-system grammar matching

As previously noted, there are algorithms which have been proposed to obtain non-parametric L-system rules, from a string of non-parametric L-system symbols [38]. So, a potential fitness function for proposed L-system inverse problem could be obtained based on the comparison of converted image into an L-system string (target string) and the string generated by GA (final string).

Every graphical structure can be converted to a final string with different algorithms [33,47,48] whose graphical interpretation results in the given appropriate structure. However, none of these algorithms can solve L-system inverse problem to obtain parametric growth rules. Farooq *et al*. [47] showed that complicated tree-like structures could be converted to a final parametric string by a multilayered architecture of GA. They also examined the impact of different GA parameters, selection methods and the objective functions used as their calculated fitness function in GA on their algorithm efficiency.

Chuai-Aree *et al*. [48] developed a straightforward algorithm to achieve parametric L-system final string. This algorithm starts with reading the input image for two dimensions or sliced images for three dimensions. Anisotropic diffusion filtering is applied as the preprocessing step. Either region or volume growing technique is conducted by the set of given initial starting points. Thinning and skeletonization are the next steps respectively. At the end, L-system final string is generated and ready for further uses [48]. In this work, this straightforward algorithm was used to convert a two-dimensional target structure to a target string.

Although XOR comparison of target string against the string generated by GA can be helpful as the fitness function, it will fail in some situations in parametric L-systems. For example, consider the following two simple L-system strings:

4.3 $$\begin{array}{l} a.\;F(100)\\ b.\;F(25)+(0)F(25)+(0)F(25)+(0)F(25). \end{array}\}$$

A simple line with a length of 100 is the graphical interpretation of both strings, but XOR comparison of alphabets of two strings leads to an inappropriate score. Accordingly, an inclusion of both graphical and grammatical criteria must be considered to define a proper fitness function.

#### 4.1.4. Hybrid fitness function

In this work, a combination of feature, grammar and pixel-by-pixel matching was used to calculate the difference score between two structures. In order to have a more accurate comparison, each structure was split into its components. Nested brackets which represent branches in L-system were considered as components, and ‘*η*’ is the number of branches. So the comparison is made between the target and simulated structures with the feature, grammar and pixel-by-pixel matching in local and global manners. Fitness function was defined as

4.4 $$f=\overset{\eta }{\underset{i=1}{\sum }}g_{H,i}(\text{st}{\text{r}}_{t}\text{, st}{\text{r}}_{s})+\overset{\eta }{\underset{i=1}{\sum }}g_{G,i}(\text{st}{\text{r}}_{t}\text{, st}{\text{r}}_{s})+\overset{\eta }{\underset{i=1}{\sum }}g_{P,i}(\text{st}{\text{r}}_{t}\text{, st}{\text{r}}_{s}),$$

where $g_{H}(\text{st}{\text{r}}_{t}\text{, st}{\text{r}}_{s})$ calculates mean Hamming distance between matched keypoints of the target and simulated structures which were detected and described with ORB algorithm. $g_{G}(\text{st}{\text{r}}_{t}\text{, st}{\text{r}}_{s})$ computes grammatical differences between target and simulated structures as was mentioned in the previous section. Pixel-by-pixel comparison is also conducted by $g_{P}(\text{st}{\text{r}}_{t}\text{, st}{\text{r}}_{s})$. OpenCV C++ library [46] was used to implement XOR algorithm for pixel-by-pixel comparison and ORB algorithm for mean Hamming distance calculation between matched keypoints. Also, bitwise XOR operation for grammar comparison was implemented in our C++ platform [39].

Terms of the fitness function were normalized to lie in the range of [0,1]. The normalization was conducted as follows:

4.5 $$\begin{array}{rl} {\hat{g}}_{H}(\text{st}{\text{r}}_{t}\text{, st}{\text{r}}_{s}) & \;=\frac{g_{H}(\text{st}{\text{r}}_{t}\text{, st}{\text{r}}_{s})}{\tau },\\ {\hat{g}}_{G}(\text{st}{\text{r}}_{t}\text{, st}{\text{r}}_{s}) & \;=\frac{g_{G}(\text{st}{\text{r}}_{t}\text{, st}{\text{r}}_{s}\text{) }}{\alpha }\\ \text{and}\;{\hat{g}}_{P}(\text{st}{\text{r}}_{t}\text{, st}{\text{r}}_{s}) & \;=\frac{g_{P}(\text{st}{\text{r}}_{t}\text{, st}{\text{r}}_{s})}{\upsilon }, \end{array}\}$$

in which *τ* is Hamming distance threshold, *α* is the maximum length of L-system string allowed in the algorithm, and *υ* is the summation of non-white pixels in target and simulated images as the maximum possible pixel-by-pixel comparison difference between them. Fitness function was modified to

4.6 $$\begin{array}{l} f=\overset{\eta }{\underset{i=1}{\sum }}\omega_{1}.{\hat{g}}_{H,i}(\text{st}{\text{r}}_{t}\text{, st}{\text{r}}_{s})+\overset{\eta }{\underset{i=1}{\sum }}\omega_{2}.{\hat{g}}_{G,i}(\text{st}{\text{r}}_{t}\text{, st}{\text{r}}_{s})+\overset{\eta }{\underset{i=1}{\sum }}\omega_{3}.{\hat{g}}_{P,i}(\text{st}{\text{r}}_{t}\text{, st}{\text{r}}_{s})\\ \omega_{1}+\omega_{2}+\omega_{3}=1, \end{array}\}$$

where *ω*_1_, *ω*_2_ and *ω*_3_ are weight factors that should be defined properly for each case. The weight factors can affect optimization convergence and run time. In this work, weight factors were obtained via a trial and error procedure, but their optimum values can be found through another optimization process. The following illustrative example is presented to show the calculation procedure.

Example 4.2. For calculation of the fitness function between three structures introduced in example 4.1, the first step is splitting them into their components. Owing to the symmetry of the structures, just three components out of seven were selected to calculate the fitness function (table 2).

**Table 2.** Mean Hamming and grammar distances between components shown in figure 5.

mean Hamming distance		grammar distance		pixel-by-pixel comparison	
${\hat{g}}_{H}(C_{a}^{1},C_{b}^{1})=0.33$	${\hat{g}}_{H}(C_{a}^{1},C_{c}^{1})=0.36$	${\hat{g}}_{G}(C_{a}^{1},C_{b}^{1})=0.56$	${\hat{g}}_{G}(C_{a}^{1},C_{c}^{1})=0$	${\hat{g}}_{P}(C_{a}^{1},C_{b}^{1})=0.23$	${\hat{g}}_{P}(C_{a}^{1},C_{c}^{1})=0.54$
${\hat{g}}_{H}(C_{a}^{2},C_{b}^{2})=0.25$	${\hat{g}}_{H}(C_{a}^{2},C_{c}^{2})=0.33$	${\hat{g}}_{G}(C_{a}^{2},C_{b}^{2})=0.64$	${\hat{g}}_{G}(C_{a}^{2},C_{c}^{2})=0$	${\hat{g}}_{P}(C_{a}^{2},C_{b}^{2})=0.28$	${\hat{g}}_{P}(C_{a}^{2},C_{c}^{2})=0.35$
${\hat{g}}_{H}(C_{a}^{3},C_{b}^{3})=0.22$	${\hat{g}}_{H}(C_{a}^{3},C_{c}^{3})=0.36$	${\hat{g}}_{G}(C_{a}^{3},C_{b}^{3})=0.89$	${\hat{g}}_{G}(C_{a}^{3},C_{c}^{3})=0$	${\hat{g}}_{P}(C_{a}^{3},C_{b}^{3})=0.4$	${\hat{g}}_{P}(C_{a}^{3},C_{c}^{3})=0.29$

**Figure 5.**
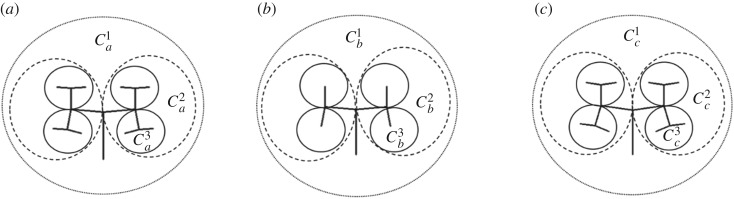
Components of L-systems defined in example 4.1: (*a*) 3 growth cycles and *δ* = 85°, (*b*) 2 growth cycles and *δ* = 85° and (*c*) 3 growth cycles and *δ* = 82°.

The threshold (*τ*) was considered 40 as mentioned by Khan *et al*. [42]. *α* was assumed as the length of target string (figure 5*a*). Furthermore, *ω*_1_, *ω*_2_ and *ω*_3_ were all assumed to be 1/3.

Considering all components:

4.7 $$\begin{array}{rl} f_{a,b} & \;=3.17\\ \text{and}\;f_{a,c} & \;=1.62, \end{array}\}$$

where $f_{a,b}$ and $f_{a,c}$ are differences between figure 5*a*,*b*, and figure 5*a–c* according to equation (4.6). Equation (4.7) shows that figure 5*a* and *c* have lower distance than figure 5*a* and *b* with this hybrid fitness function and proposed weight factors. In this work, if there is not a good match between two structures or there is no branch in a zone, mean Hamming distance was considered equal to the threshold. So, in example 4.2 mean Hamming distance between $C_{a}^{3}$ and $C_{b}^{3}$ is equal to 1 instead of 0.22 in our main algorithm. The reason is that it is not a good idea to match a single line to a linear structure.

### 4.2. Decision variables

As was mentioned in §4.1, most of the tree-like structures can be derived from equation (4.2). Due to the generality of equation (4.2), it was used to simulate retinal vasculature structure. All possible decision variables based on equation (4.2) are presented in table 3.

**Table 3.** Decision variables.

decision variable	data structure
*m*, *n*	integer number
$z_{0},\theta_{0}$	real number
$T,T^{\prime },D$	character
g(Z,Θ),f(Z,Θ)	parameter functions including characters and numbers

Here, we are also looking for f(Z,Θ) and g(Z,Θ) which are two unknown functions that show the relationship between length and angle of predecessor and successor, respectively. f(Z,Θ) and g(Z,Θ) are called parameter functions.

Due to different data structures, we have a MINLP problem. In this work, GA was employed to obtain the best L-system rules and parameters for a given structure.

## 5. Genetic algorithm

GAs are among the most powerful evolutionary algorithms that have been well addressed in the literature [49]. Defining an appropriate genetic representation, operators, and fitness function are the main steps to solve an optimization problem using GA. In the present work, an open source C++ framework called GAlib [50] was employed to solve L-system inverse problem. The fitness function and its corresponding decision variables were described in the previous section.

As mentioned earlier, our optimization problem consists of various types of decision variables including character, integer and real data types. In GAlib, GAAlleleSet is a container that can involve enumerated, bounded, or bounded with discretization sets of data types [50]. So, a composite chromosome or genome composed of GAAlleleSets of int, real and char data types was employed as the genetic representation (i.e. genotypes) of our decision variables (i.e. phenotypes). It should be noted that in order to avoid getting physically meaningless genotypes (i.e. those for which there are no physically meaningful decision variables), each AlleleSet has its own genetic operators in GALib and this is one of the great features of GALib that we have used. In this work, steady-state GA was used for optimization. In this algorithm, a temporary population of individuals was created and added to the previous population. Then, the worst individuals were removed based on roulette wheel selection scheme to achieve a population with the original size. UniformInitializer, FlipMutator and UniformCrossover defined in GAlib [50] were used as the genetic operators.

## 6. Method

**Figure 6.**
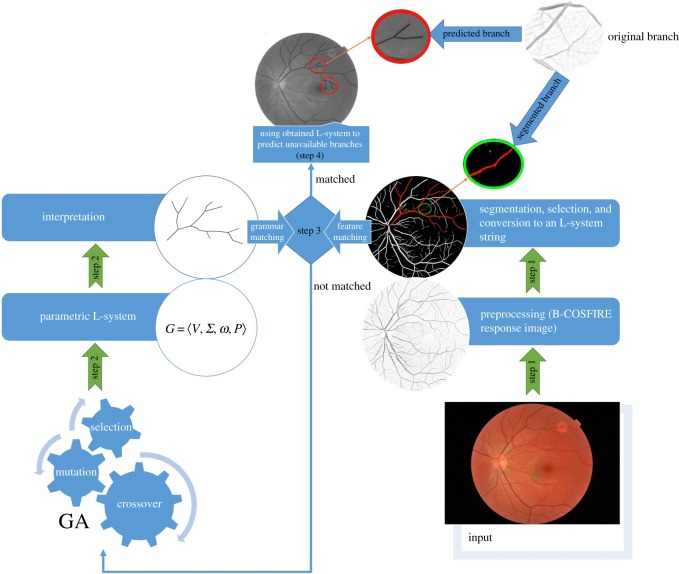
The framework flowchart.

Figure 6 shows the flowchart of the procedure according to which various tentative L-system was used to obtain its corresponding retinal vascular structure and its fitness function. In the first step, target image should be preprocessed and converted to one L-system string. Automatic segmentation of blood vessel branches based on Combination of Shifted Filter Responses (COSFIRE), proposed by Azzopardi *et al*. [51], is used to extract blood vessels from original fundus image. Extracted branches obtained from segmentation are then converted to an L-system string using algorithm proposed by Chuai-Aree *et al*. [48]. The obtained L-system string is then compared against the one obtained by simulated structure resulting from GA in the second step, as mentioned in §4. Parameters in each growth cycle are calculated with simple linear functions. In the third step, the existence of general nonlinear functions for each parameter is investigated. Finally, unavailable branches would be predicted based on the obtained general nonlinear functions.

To illustrate the details of the proposed algorithm, it would be implemented step by step for the following case study.

*Illustrative case study.* In this case study, existence of simple rewriting rule for the structure presented by Kókai *et al*. [33] is investigated.

### 6.1. Step 1: Preprocessing and converting target structure to one L-system string

Owing to the lack of information about original image and inconsistency between given image scale and given L-system parameters, the image was preprocessed, and final L-system string was regenerated with the algorithm presented by Chuai-Aree *et al*. [48].

6.1 $$\begin{array}{rl} & F(24)[+(35)F(16)[+(24)F(10)+(14)F(10)+\\ & \;(6)F(18)[+(47)F(25)][-(11)F(20)]][-(10)F(18)+(26)F(15)[-(6)F(10)+\\ & \;(34)F(16)+(32)F(12)][-(43)F(24)]]][F(32)[-(7)F(8)+(43)F(24)][-(14)F(26)]]. \end{array}$$

### 6.2. Step 2: Solving L-system inverse problem

To solve L-system inverse problem for this structure, the problem was formulated in terms of equation (4.1). Equation (4.2) was simplified as follows:

6.2 $$\begin{array}{l} \omega :A(z_{0},\theta_{0})\\ p_{1\ldots n}:A(Z,\Theta )\to M_{1}^{0}[M_{1}^{1}M_{2}^{1}][M_{1}^{2}M_{2}^{2}]\\ M_{m,n}^{b}={\left(D^{b}(g^{b}(Z,\Theta ))T^{b}(f^{b}(Z,\Theta ))\right)}_{1\ldots m,1\ldots n}. \end{array}\}$$

Further, it was assumed that the axiom length and angle are equivalent to those for the main root of the target structure. In this case, the length of the main root and its angle relative to the axis X are 24 and 15, respectively. So, we have

6.3 ω:A(24,15).

On the other hand, it is assumed that $g^{0}(Z,\Theta )=Z$. Based on described assumptions, $M_{m,n}^{b}$ are the only decision variables. The only remaining problem is to define g(Z,Θ),f(Z,Θ) format.

In our algorithm, g(Z,Θ),f(Z,Θ) was considered to be a simple linear function as bZ+cΘ. In every rewriting step, different constants would be considered by GA for these functions. Using this method, complicated functions can be approximated with linear ones at each growth cycle.

Following equations show one of the possible paths that could lead to the solution:

6.4 $$\begin{array}{l} \text{Growth}\;\text{cycle }0\\ A(24,15). \end{array}\}$$

For the sake of brevity, the following rules resulting from optimization process have been suggested as candidate rules:

6.5 $$\begin{array}{rl} & A(Z,\Theta )\to F(z)[+(g_{1}(Z,\Theta ))F(g_{2}(Z,\Theta ))+(g_{3}(Z,\Theta ))A(g_{4}(Z,\Theta ))][-(g_{5}(Z,\Theta ))F(g_{6}(Z,\Theta ))\;+\\ & \;(g_{7}(Z,\Theta ))A^{\prime }(g_{8}(Z,\Theta ))] \end{array}$$

and

6.6 $$\begin{array}{rl} & A^{\prime }(Z,\Theta )\to F(z)[+(g_{1}(Z,\Theta ))F(g_{2}(Z,\Theta ))+(g_{3}(Z,\Theta ))F(g_{4}(Z,\Theta ))][-(g_{5}(Z,\Theta ))F(g_{6}(Z,\Theta ))\;+\\ & \;(g_{7}(Z,\Theta ))F(g_{8}(Z,\Theta ))]. \end{array}$$

The rewriting rules for both *A* and *A′* are the same, other than the fact that the one corresponding to *A′* is applied once because it does not contain any *A* or *A′* to be rewritten at the next growth cycle.

It was also assumed that length sizes are equal in each block, Hence

6.7 $$\begin{array}{rl} g_{2}(Z,\Theta ) & \;=g_{4}(Z,\Theta )\\ \text{and}\;g_{6}(Z,\Theta ) & \;=g_{8}(Z,\Theta ). \end{array}\}$$

As it was mentioned, linear functions would be replaced in the rule for each rewriting procedure. For example, GA result for the first growth cycle is

6.8 $$A(Z,\Theta )\overset{\;}{\to }F(z)\left[+(2\Theta )F\left(\frac{Z}{3}-0.5\right)+\left(\frac{\Theta }{5}\right)A\left(\frac{Z}{3}-0.5\right)\right]\left[-\left(\frac{\Theta }{3}\right)F\left(\frac{2Z}{3}\right)+\left(\frac{\Theta }{3}\right)A^{\prime }\left(\frac{2Z}{3}\right)\right].$$

So,

6.9 $$\begin{array}{l} \text{Growth cycle }1\\ F(24)[+(30)F(7.5)+(3)A(7.5)][-(8)F(16)+(10)A^{\prime }(16)]. \end{array}\}$$

For the sake of brevity, only numerical parameters resulting from different linear functions will be reported afterward:

6.10 $$\begin{array}{l} \text{Growth}\;\text{cycle }2\\ F(24)[+(30)F(7.5)+(3)F(7.5)[+(35)F(20)+(10)A(20)][-(10)F(18)+\\ (25)A^{\prime }(18)]][-(8)F(16)+(10)F(16)[+(20)F(16)+(18)F(16)][-(22)F(12.5)+\\ \left(5.5)F(12.5)]\right] \end{array}\}$$

and

6.11 $$\begin{array}{l} \text{Growth cycle 3}\\ F(24)[+(30)F(7.5)+(3)F(7.5)[+(35)F(20)+(10)F(20)[+(50)F(12.5)+\\ (4)A(12.5)][-(10)F(10)+(3)A^{\prime }(10)]][-(10)F(18)+(25)F(18)[+(12)F(18)+\\ (45)F(18)][-(45)F(10)+(0.01)F(10)]]][-(8)F(16)+(10)F(16)[+(20)F(16)+\\ (18)F(16)][-(22)F(12.5)+(5.5)F(12.5)]]. \end{array}\}$$

After three growth cycles, L-system final string will be compared against the one representing target structure with the hybrid fitness function. If the difference between simulated and target structures is acceptable, the optimization process is terminated. The values of GA parameters are given in table 4.

**Table 4.** Parameters of genetic algorithm.

population size	30
crossover rate	0.9
mutation rate	0.03
number of generation	500

**Figure 7.**
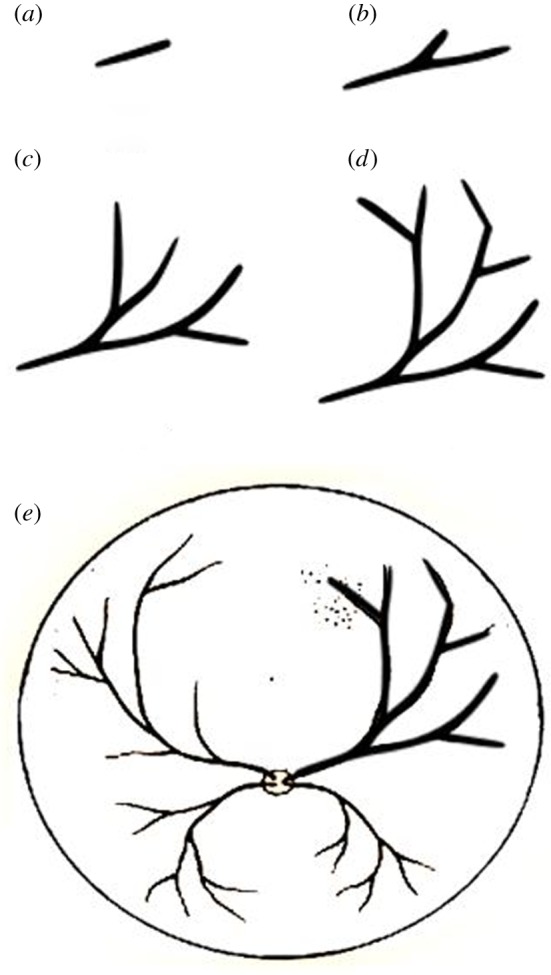
Graphical interpretation of (*a*) 0, (*b*) 1, (*c*) 2 and (*d*) 3 growth cycles of simulated structure and (*e*) graphical matching of simulated and target structures.

Final graphical string interpretation for each growth cycle and a comparison with the target image are shown in figure 7.

The result obtained by the proposed method and its corresponding reference one are compared in figure 7*e*. This figure shows that simulated and target structures are matched perfectly. To express the results quantitatively based on equation (4.4), the pixels that belong to the branch in the target image and that are simulated correctly are counted as true positives (TP), otherwise they are counted as false negatives (FN). The pixels that belong to the background and that are simulated as branch are counted as false positives (FP), otherwise they are counted as true negatives (TN). Accuracy, sensitivity and specificity are well-known metrics for pixel-by-pixel comparison [14] defined as follows:

6.12 $$\begin{array}{rl} \text{Acc} & \;=\frac{\text{TP}+\text{TN}}{\text{TP}+\text{TN}+\text{FP}+\text{FN}},\\ \text{Se} & \;=\frac{\text{TP}}{\text{TP}+\text{FN}}\\ \text{and}\;\text{Sp} & \;=\frac{\text{TN}}{\text{TN}+\text{FP}}. \end{array}\}$$

The values of accuracy, sensitivity, specificity, ${\hat{g}}_{H}(\text{st}{\text{r}}_{t}\text{, st}{\text{r}}_{s})$, ${\hat{g}}_{G}(\text{st}{\text{r}}_{t}\text{, st}{\text{r}}_{s})$, and ${\hat{g}}_{P}(\text{st}{\text{r}}_{t}\text{, st}{\text{r}}_{s})$, resulting from comparison of target and simulated branches, are provided in table 5.

**Table 5.** Quantitative results for illustrative case study.

accuracy	0.99
specificity	0.99
sensitivity	0.68
${\hat{g}}_{P}(\text{st}{\text{r}}_{t}\text{, st}{\text{r}}_{s})$	0.47
${\hat{g}}_{H}(\text{st}{\text{r}}_{t}\text{, st}{\text{r}}_{s})$	0.11
${\hat{g}}_{G}(\text{st}{\text{r}}_{t}\text{, st}{\text{r}}_{s})$	0.22

In the next step, it is tried to find general functions for each *g*_1_ to *g*_8_ to reproduce the same results generated by linear functions in rewriting process. This is done by finding a correlation between the lengths and angles of the predecessor and successor.

### 6.3. Step 3: Searching for general parameter functions

In correlation step, angles were described in radians. Also, $\bar{Z}$ was defined as a dimensionless variable:

6.13 $$\bar{Z}=\frac{Z}{L_{m}-L_{0}},$$

where *Z* is the length of the line, *L_m_* is the distance between initiation point and the farthest point in the structure and *L*_0_ is the distance between initiation point and the centre of the line.

All inputs and outputs related to each of the functions $g_{1..8}$ in equation (6.5) were collected similar to what is shown in table 6 for $g_{4}(Z,\Theta )$. Nonlinear regression was used to find and fit general functions.

**Table 6.** Input–output data related to $g_{4}(Z,\Theta )$.

$Z_{\mathrm{predecessor}}$		$\Theta_{\mathrm{predecessor}}$ ^a^		$Z_{\mathrm{successor}}(L)$	
(*Z*)	$\left(\bar{Z}\right)$	(degree)	(radian)	(*Z*)	$\left(\bar{Z}\right)$
24	0.28	15	0.26	7.5	0.12
7.5	0.12	48	0.84	20	0.55
20	0.55	93	1.62	12.5	0.44
16	0.34	17	0.30	16	0.87
18	0.59	63	1.10	18	2.21

^a^A line angle is defined as its angle relative to the axis *X*.

Correlation information for $g_{4}(\bar{Z},\Theta )$ is shown in table 7.

**Table 7.** Regression information for $g_{4}(Z,\Theta )$.

$g_{4}(\bar{Z},\Theta ):a+b\bar{Z}+c\ln(\Theta )+d\ln{\left(\Theta \right)}^{2}$		$R^{2}=0.99$	
variable	value	standard error	Prob(t)
*a*	−0.42	7 × 10^−3^	0.010
*b*	4.93	1.7 × 10^−2^	0.002
*c*	−2.61	1.1 × 10^−2^	0.002
*d*	−2.41	9 × 10^−3^	0.002

Correlations for all other parameter functions are shown in table 8. As the result, the structure presented by Kókai *et al*. [33] can be reproduced with the following simple rule:

6.14 $$\begin{array}{l} \omega :\;A(24,15)\\ p_{1}:\;A(Z,\Theta )\to F(z)[+(g_{1}(Z,\Theta ))F(g_{2}(Z,\Theta ))+(g_{3}(Z,\Theta ))A(g_{4}(Z,\Theta ))]\\ \;\;\;\;\;\;\;\;\;\;[-(g_{5}(Z,\Theta ))F(g_{6}(Z,\Theta ))+(g_{7}(Z,\Theta ))A^{\prime }(g_{8}(Z,\Theta ))]. \end{array}\}$$

**Table 8.** Coefficients of functions presented in equation (6.13).

	$g(\bar{Z},\Theta ):a+b\bar{Z}+c\ln(\Theta )+d\ln{\left(\Theta \right)}^{2}$				
	*a*	*b*	*c*	*d*	*R*^2^
$g_{1}(\bar{Z},\Theta )$	0.94 ± 0.03	−1.41 ± 0.08	1.06 ± 0.05	0.79 ± 0.04	0.99
$g_{3}(\bar{Z},\Theta )$	−0.20 ± 0.02	1.85 ± 0.06	−1.06 ± 0.03	−0.94 ± 0.03	0.99
$g_{5}(\bar{Z},\Theta )$	−0.168 ± 0.001	1.771 ± 0.004	−0.883 ± 0.002	−0.763 ± 0.002	0.99
$g_{7}(\bar{Z},\Theta )$	0.562 ± 0.005	−0.96 ± 0.01	0.056 ± 0.008	−0.044 ± 0.007	0.99
$g_{8}(\bar{Z},\Theta )$	0.387 ± 0.002	1.100 ± 0.005	−0.504 ± 0.003	−0.554 ± 0.002	0.99

It should be noted that based on equation (6.7), $g_{2}(Z,\Theta )=g_{4}(Z,\Theta )$ and $g_{6}(Z,\Theta )=g_{8}(Z,\Theta )$.

### 6.4. Step 4: Predicting unavailable branches with suggested general functions

**Figure 8.**
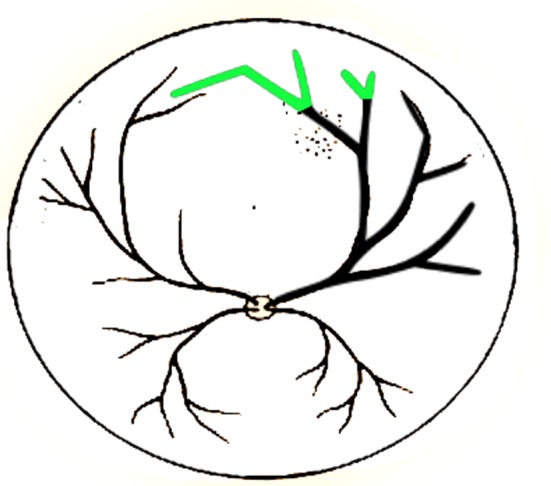
Predicted branches for illustrative case study using suggested general functions.

Applying suggested general functions, one more rewriting procedure was conducted on equation (6.14). Prediction results are shown in figure 8 with green lines.

## 7. Results and discussion

There are lots of Web-based fundus image galleries obtained through various ophthalmic imaging systems [52–54]. After going through the details of the proposed method for the development of retinal vascular structure with an illustrative case study, to show the accuracy and flexibility of the proposed method, it is used to develop the retina vascular structure based on practical images obtained from various imaging techniques.

**Figure 9.**
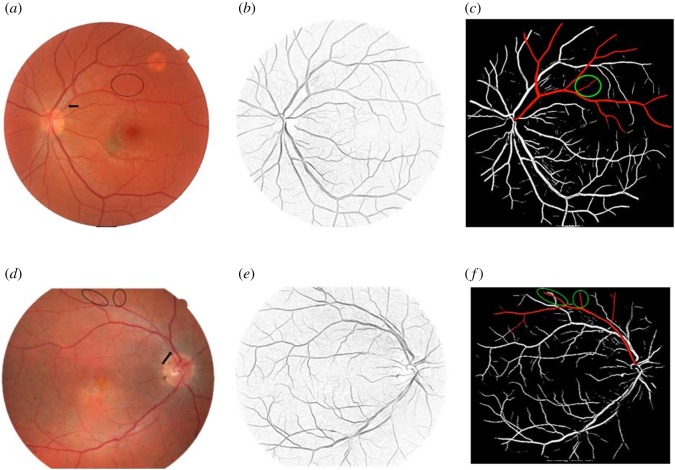
Thirty-year-old woman's left eye: (*a*) original fundus image, (*b*) B-COSFIRE filter response image, (*c*) segmented retinal blood vessels. Forty-nine-year-old man's right eye: (*d*) original fundus image [54], (*e*) B-COSFIRE filter response image, (*f*) segmented retinal blood vessels.

To investigate the similarity of growth rules of retinal vasculature in different eyes, 108 fundus images were analysed by the algorithm presented in this work. For instance, two fundus images along with their preprocessed and segmented images are shown in figure 9.

Branches shown by arrows in figure 9*a*,*d* were found to follow the same growth rule. Due to the lack of information about the circled branches, they would be predicted in the next section. For the sake of brevity, simplified branching rule will be presented in this section.

The proposed algorithm in the previous section was implemented, and the axioms and rewriting rules which are shown by equation (7.1) were obtained:

7.1 $$\begin{array}{rl} & \omega_{\mathrm{for}\;(a)\;\mathrm{in}\;\mathrm{figure}\;8}:\;F(60,44.5)+(0)A(60,44.5)\\ & \omega_{\mathrm{for}\;(b)\;\mathrm{in}\;\mathrm{figure}\;8}:\;F(80,123)+(0)A(80,123)\\ & p_{1}:\;A(Z,\Theta )\to F(z)[\pm (g_{1}(Z,\Theta ))F(f_{1}(Z,\Theta ))\mp (g_{2}(Z,\Theta ))B(f_{2}(Z,\Theta ))]\\ & \;\;\;\;\;\;\;\;\;[\mp (g_{3}(Z,\Theta ))F(f_{3}(Z,\Theta ))\pm (g_{4}(Z,\Theta ))A(f_{4}(Z,\Theta ))]\\ & p_{2}:\;B(Z,\Theta )\to F(z)[\pm (g_{5}(Z,\Theta ))F(f_{5}(Z,\Theta ))\mp (g_{6}(Z,\Theta ))F(f_{6}(Z,\Theta ))]\\ & \;\;\;\;\;\;\;\;\;[\mp (g_{7}(Z,\Theta ))F(f_{7}(Z,\Theta ))\pm (g_{8}(Z,\Theta ))F(f_{8}(Z,\Theta ))], \end{array}\}$$

where the first axiom and first rule correspond to the L-system representing figure 9*a* and the second ones reflects the L-system representing figure 9*b*. The following parametric strings are the final best matched strings resulting from the proposed algorithm after four L-system growth cycles:

7.2 $$\begin{array}{l} \text{For}\;\text{figure}\;9a\\ F(60)+(0)F(60)[+(58)F(60)-(23)F(65)[+(42.5)F(48)-(10)F(60)][-(22)F(65)+\\ (5)F(80)]][-(30)F(21)+(1)F(77)[+(27)F(80)-(-7)F(81)[+(24.5)F(37)-\\ (34)F(56)][-(35)F(60)+(1)F(75)]][-(33)F(34)+(9)F(8)[+(X)F(X)-\\ (X)F(X)[+(X)F(X)-(X)F(X)][-(X)F(X)+(X)F(X)]][-(30)F(36)+\\ (30)F(52)[+(10)F(63)-(6.5)F(105)[+(26)F(45)-(1.5)F(40)][-(55)F(62)+\\ (40)F(80)]][-(55)F(57)+(37)F(6)[+(20)F(18)-(7)+B(30)][-(45)F(31.5)+\\ \left(-3)A(27)]]]]\right] \end{array}\}$$

and

7.3 $$\begin{array}{l} \text{For}\;\text{figure}\;9b\\ F(80)+(0)F(80)[-(44.5)F(50)+(5)F(60)[-(7)F(28)+(17)F(30)][+(40)F(45)-\\ (12)F(45)]][+(30)F(47)-(4.5)F(85)[-(X)F(X)+(X)F(X)[-(X)F(X)+\\ (X)F(X)][+(X)F(X)-(X)F(X)]][+(21)F(40)-(3)F(35)[-(X)F(X)+(X)F(X)[-(X)F(X)+\\ (X)F(X)][+(X)F(X)-(X)F(X)]][+(24)F(77.5)-(5.5)F(100)[-(9)F(55)+\\ (-5)F(20)[-(30)F(40)+(-15)F(60)][+(18)F(51)-(2.5)F(60)]][+(83)F(34)-\\ (1)F(2)[-(55)F(11)+(0)B(10)][+(32)F(14)-(0)A(9)]]]]]. \end{array}\}$$

The parameters shown by *X* correspond to the circled branches of figure 9 which should be predicted due to the lack of information.

Values of various parameters in equations (7.2) and (7.3) have all been correlated with a set of general functions representing $g_{i}$ and $f_{i}$ (*i* = 1,2, … 8), respectively. The obtained correlating functions for both cases (i.e. those represented by figure 9*a*,*b*) were compared to investigate the generalizability of the obtained correlating functions. To obtain general functions, Θ was considered as the angle relative to the axis *Y* described in radians. Also, two dimensionless variables were defined in the form of

7.4 $$\begin{array}{rl} {\bar{Z}}_{\mathrm{predecessor}} & \;=\frac{Z_{\mathrm{predecessor}}}{L_{0}}\\ \text{and}\;{\bar{Z}}_{\mathrm{successor}} & \;=\frac{Z_{\mathrm{successor}}}{Z_{\mathrm{predecessor}}}, \end{array}\}$$

where *Z*_predecessor_ is the length of predecessor, *Z*_successor_ is the length of successor and *L*_0_ is the distance between initiation point and the centre of predecessor.

The simulations and analysis of the existing cases in our database based on the mentioned procedure led to an astonishing result. Not only do the formats of all general functions describing the lengths ($f_{1..8}$) and angles ($g_{1..8}$) share the same formats for each case but also the formats and even the coefficients of general functions for couple of cases particularly those shown in figure 9*a*,*b* are the same. Table 9 presents this similarity for two typical functions (i.e. *f*_1_ and *g*_2_) for cases shown in figure 9*a*,*b*.

**Table 9.** Instances of general functions for two cases shown in figure 9*a*,*b*.

$f_{1}(\bar{Z},\Theta )=a_{1}b_{1}^{\Theta }{\bar{Z}}^{c_{1}}$					
	*a*_1_	*b*_1_	*c*_1_	*d*_1_	$R_{1}^{2}$
figure 9*a*	1.2±0.6	0.3±0.1	−1.0±0.2	—	0.99
figure 9*b*	1.4±0.2	0.05±0.02	−1.9±0.3	—	0.99

g2(Z¯,Θ)=a2+b2Θ+c2ln⁡(Θ)2+d2ln⁡(Z¯)∫					
	*a*_2_	*b*_2_	*c*_2_	*d*_2_	R2∫2
figure 9*a*	5.1±0.6	−5±1	−0.015±0.007	−1.3±0.4	0.92
figure 9*b*	4.90±0.04	−4.9±0.3	−0.030±0.008	−1.9±0.2	0.98

To show the correlation between (${\bar{Z}}_{\mathrm{predecessor}}$, $\Theta_{\mathrm{predecessor}}$) and (${\bar{Z}}_{\mathrm{successor}}$, $\Theta_{\mathrm{successor}}$) of all ten similar cases [52] along with figure 9*a*,*b*, coefficients of obtained general functions have been reported in table 10. Regression coefficients can be updated for each case such as those shown in table 9.

**Table 10.** Coefficients of functions presented in equation (6.14).

	$a_{i}$	$b_{i}$	$c_{i}$	$d_{i}$	$R_{i}^{2}$
fi(Z¯,Θ)=aibiΘZ¯ci∫					
$f_{1}(\bar{Z},\Theta )$	1.0±0.1	0.24±0.05	−1.1±0.1	—	0.98
$f_{2}(\bar{Z},\Theta )$	2.6±0.8	0.09±0.05	−1.4±0.3	—	0.89
$f_{3}(\bar{Z},\Theta )$	0.7±0.2	0.9±0.2	−0.4±0.1	—	0.93
$f_{4}(\bar{Z},\Theta )$	2.1±0.6	0.3±0.1	−0.8±0.2	—	0.85
$f_{5}(\bar{Z},\Theta )$	0.15±0.05	0.8±0.2	−0.9±0.2	—	0.90
$f_{6}(\bar{Z},\Theta )$	0.15±0.03	0.7±0.3	−1.0±0.2	—	0.92
$f_{7}(\bar{Z},\Theta )$	0.24±0.07	0.7±0.2	−0.8±0.2	—	0.88
$f_{8}(\bar{Z},\Theta )$	0.31±0.09	0.9±0.2	−0.7±0.1	—	0.89
$g_{i}(\bar{Z},\Theta )=a_{i}+\frac{b_{i}}{\Theta }+\frac{c_{i}}{\ln{\left(\Theta \right)}^{2}}+\frac{d_{i}}{\ln(\bar{Z})}$					
$g_{1}(\bar{Z},\Theta )$	3.6±0.5	−4.5±1.1	−0.022±0.008	−1.2±0.2	0.98
$g_{2}(\bar{Z},\Theta )$	4.9±0.1	−5.2±0.6	−0.03±0.01	−2±0.5	0.91
$g_{3}(\bar{Z},\Theta )$	3.3±0.7	−4±1	−0.05±0.02	−2.3±0.3	0.89
$g_{4}(\bar{Z},\Theta )$	5.1±0.1	−6.1±0.1	−0.03±0.01	−2.4±0.3	0.87
$g_{5}(\bar{Z},\Theta )$	2.5±0.1	−0.95±0.03	−0.013±0.001	−0.4±0.1	0.96
$g_{6}(\bar{Z},\Theta )$	3.1±0.1	−0.75±0.05	−0.011±0.002	−0.15±0.08	0.87
$g_{7}(\bar{Z},\Theta )$	2.4±0.1	−0.98±0.01	−0.015±0.001	−0.71±0.01	0.98
$g_{8}(\bar{Z},\Theta )$	3.1±0.1	−0.77±0.03	−0.013±0.001	−0.09±0.05	0.85

**Figure 10.**
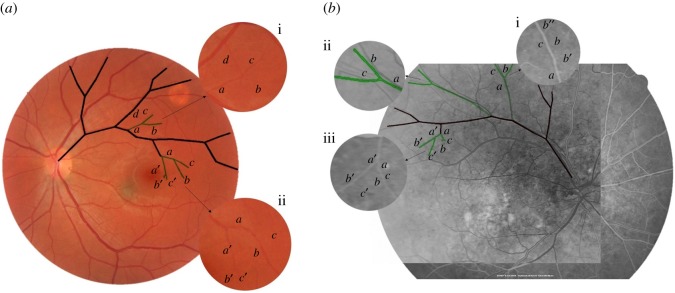
Comparison between simulated and predicted branches with target structures presented in (*a*) figure 9*a* and (*b*) figure 9*d*.

The general functions were used to predict branches shown in circled zones in figure 9. Also with the help of these functions, one more growth cycle was conducted on equations (7.2) and (7.3) to predict next generation branches that could not be recognized due to image resolution. Simulated and predicted branches are shown in figure 10.

In order to compare circled branches in figure 9*b* with predicted ones, wide-field fundus image of figure 9*b* [55] is provided in figure 10*b*. Black lines represent simulated structures. Green lines show predicted branches based on suggested general functions. Accuracy, sensitivity, specificity and fitness function values for simulated and predicted branches in both cases are given in table 11. As mentioned in §6, COSFIRE algorithm was used to extract blood vessels in the first step. Performance of the COSFIRE algorithm which was used in the first step of proposed algorithm affects the results of simulation performance with regard to accuracy, specificity and selectivity metrics. As shown in table 11, the accuracy and specificity are not changed significantly compared to the segmentation step (COSFIRE results). Sensitivities of the simulated and predicted results are far less than segmentation result due to the limitations imposed by obtained growth rules. However, unlike image processing methods, the proposed algorithm has the capability to obtain the structure growth rules, if any exist.

**Table 11.** Quantitative results for figure 10.

	figure 10*a*			figure 10*b*		
	COSFIRE [51]	simulated	predicted	COSFIRE [51]	simulated	predicted
accuracy	0.98	0.91	0.93	0.98	0.90	0.93
specificity	0.99	0.95	0.96	0.99	0.95	0.97
sensitivity	0.92	0.57	0.67	0.90	0.51	0.56
${\hat{g}}_{P}(\text{st}{\text{r}}_{t}\text{, st}{\text{r}}_{s})$	—	0.58	0.50	—	0.63	0.58
${\hat{g}}_{H}(\text{st}{\text{r}}_{t}\text{, st}{\text{r}}_{s})$	—	0.21	0.13	—	0.33	0.16
${\hat{g}}_{G}(\text{st}{\text{r}}_{t}\text{, st}{\text{r}}_{s})$	—	0.31	0.48	—	0.38	0.53

It can be seen from figure 10 and table 11 that simulated and target branches are matched perfectly. Prediction results for each zone are discussed below.

Figure 10*a*

(i) It seems branches *a*, *b* and *c* are well predicted by general functions. It is clear from the image that branch *d* is related to another vessel, but the most of automated segmentation algorithms will fail to distinguish between *abc* and *d*. As is shown, predicted branch does not contain branch *d*, so our method can be useful for these situations.
(ii) Next generation of L-system defined with equation (7.2) is presented in this zone to test the ability of our method for the prediction of smaller vessels that cannot be recognized due to the lack of resolution. As can be seen in figure 10*a*(ii), it seems that $a,b,a^{\prime },b^{\prime }$ and $c^{\prime }$ are predicted appropriately. Predicted branch *c* does not have equivalent in target image due to the lack of resolution or functional error.

Figure 10*b*

(i) It seems that branches *a* and *c* are predicted well, but *b* does not have any equivalent in that position. Branch *b* is probably biased from *b*′ or $b^{\prime \prime }$ in the target image due to functional error in prediction.
(ii) In this zone, branch *a* has a little deviation from the target at the beginning and end. For this reason, *b* and *c* are biased from the real branches. Despite the deviation, the prediction is acceptable in this case.
(iii) For the next generation of L-system defined with equation (7.3), it seems that all $a,b,c,a^{\prime }$ and *b*′ are predicted well and *c*′ does not have equivalent in target image due to the lack of resolution or functional error.

It should be noted that in spite of the accuracy of the correlated functions at each growth cycle, they cannot be used for couple of consecutive growth cycles due to the error propagation and amplification. In other words, the obtained correlated functions should be used merely for prediction purposes.

## 8. Conclusion

In this work, a novel framework for the construction of retinal vasculatures has been proposed. The proposed method is based on obtaining a genetically tuned parametric L-system for a tree-like structure such as retinal vasculature. Retinal blood vessel morphology can be used to identify conditions of patients suffering from various diseases including eye disease in particular. On the other hand, L-system as a rule-based method can be useful to predict a segment of a structure that is not accessible by image processing algorithms due to the lack of information if the creation of that structure obeys the rules. Obtaining these rules from an available structure is called L-system inverse problem. The performance of the proposed method has been shown by its application for the development of retinal blood vessels of two practical case studies. It was shown that retinal blood vessels related to different people might follow the same rule which can be used to predict inaccessible parts of them. Both simulated and predicted structures matched perfectly with their corresponding target images. Prediction results improved the accuracy, sensitivity and specificity of simulated structures compared with target image. The proposed method is efficient to obtain an approximation of complicated system behaviour such as retinal vascular growth rules.

It should also be mentioned that, in some situations, deviations from target structure were seen due to the modelling error or the lack of information. Rule similarity for a group of retinal blood vessel structures related to different people can be considered as a new idea for classification of these structures. On the other hand, it is shown that searching for more accurate parameter functions based on vasculogenesis mechanism is necessary to have a better prediction.

The algorithm proposed in this work can be used to obtain parametric rules in different disease stages which affect retinal blood vessels for identification of changes in morphology of blood vessels. A richer database including both normal and abnormal fundus images with similar growth rules should be applied for further study on the role of different diseases in deviating from normal condition. Having the parametric L-system model in hand, deviation from normal width, length, and tortuosity of retinal blood vessels due to a disease such as retinopathy of prematurity [56] or diabetic retinopathy [57] can be investigated numerically.

Three-dimensional reconstruction of retinal blood vessel structure with the help of L-system and morphological properties of eye is aimed as the subject for future studies and extension of this work.

## Data accessibility

Our C++ code is deposited at GitHub [39]: https://github.com/Aaghamir/LSysInvProb.

Retinal fundus images which have similar growth rules to the proposed cases of this study are available in STARE database [52] with following numbers: im0118, im0124, im0178, im0180, im0205, im0208, im0227, im0245, im0297 and im0355: http://cecas.clemson.edu/~ahoover/stare.
